# A pooled CRISPR screen identifies the Tα2 enhancer element as a driver of *TRA* expression in a subset of mature human T lymphocytes

**DOI:** 10.3389/fimmu.2025.1536003

**Published:** 2025-03-14

**Authors:** Pascal Y. Schönberg, Ángela Muñoz-Ovalle, Maciej Paszkowski-Rogacz, Eugenia Crespo, Duran Sürün, Anja Feldmann, Frank Buchholz

**Affiliations:** ^1^ Medical Systems Biology, Faculty of Medicine Carl Gustav Carus, Technical University Dresden, Dresden, Germany; ^2^ Institute of Radiopharmaceutical Cancer Research, Helmholtz-Zentrum Dresden Rossendorf (HZDR), Dresden, Germany; ^3^ National Center for Tumor Diseases Dresden (NCT/UCC), DKFZ, Faculty of Medicine and University Hospital Carl Gustav Carus, TU Dresden University of Technology, Helmholtz-Zentrum Dresden-Rossendorf (HZDR), Dresden, Germany; ^4^ German Cancer Consortium (DKTK), Dresden, Germany

**Keywords:** TRA regulation, enhancer alpha, CRISPR screen, TCR expression, TRA locus, CAR T cells, genome editing

## Abstract

The T cell receptor (TCR) is crucial for immune responses and represents a pivotal therapeutic target for CAR T cell therapies. However, which enhancer elements drive the constitutive expression of the TCRα chain in mature, peripheral T cells has not been well defined. Earlier work has suggested that enhancer alpha is inactive in mature peripheral T cells and that an alternative enhancer element in the 5’ J region was driving *TRA* expression, while more recent findings indicated the opposite. Here, we applied a pooled CRISPR screen to probe a large genomic region proximal to the human *TRA* gene for the presence of regulatory elements. Interestingly, no sgRNA targeting the 5’ J region was identified that influenced *TRA* expression. In contrast, several sgRNAs targeting enhancer alpha element Tα2, were identified that compromised the expression of the TCRα chain in Jurkat E6.1, as well as in a subset of human primary T cells. Our results provide new insights into the regulation of *TRA* in human peripheral T cells, advancing our understanding of how constitutive *TRA* expression is driven and regulated.

## 1 Introduction

T lymphocytes are vital players in adaptive immunity, yielding unique T cell receptors (TCRs) for antigen recognition. The majority of human T cells is consisting of αβ-T cells, whose TCR is assembled from the TCRα and TCRβ chain, encoded by genes from separate genomic loci (*TRA, TRB*). In order to generate the high diversity of the TCR repertoire and to allow for functional expression, both loci are genetically rearranged by V(D)J recombination during T cell development in the thymus (1). In its unrearranged conformation, the *TRA* locus contains the *TRA*, as well as the enclosed *TRD* gene and extends over a region of 1 megabase (Mb), positioned between the olfactory receptor genes and the antiapoptosis gene DAD1 on chromosome 14 (2, 3). The biggest region is comprised of a set of 61 T cell receptor alpha joining (*TRAJ*) and 41 T cell receptor alpha variable (*TRAV*) gene segments and the T cell receptor constant (*TRAC*) domain (4). The intergenic region downstream of the last exon was found to contain the locus control region (LCR), with eight T cell specific nuclease hypersensitive (HS) domains, of which HS1 was later annotated as enhancer alpha (Eα), containing four binding sites for nuclear proteins (Tα1 - 4) (5–7).

In developing thymocytes, the process of *TRA* V(D)J recombination is initiated by the decommission of the homeodomain (HOX)A5-9 transcription factors, which repress Eα in earlier stages of T cell differentiation (8). Eα forms a CTCF/cohesin-mediated chromatin loop, bringing it in close proximity to the over 70 kilo bases (kb) distant T early α (TEA) promoter. Its de-repression allows for promoter-enhancer binding, which in turn promotes active transcription from TEA (9). As a result, the level of chromatin accessibility around recombination signal sequences (RSSs) in the *TRA* locus is increased and V(D)J recombination can be initiated by recombination activating gene (RAG)1/2 (10). To date, Eα is the only known enhancer that influences *TRA* expression (8). However, its vital role was so far mostly investigated in the context of T cell development. The factors driving constitutive expression of rearranged *TRA* in mature human T cells have only been sparsely investigated.

In one of these investigations, a transgenic reporter mouse model was generated, where the expression of hCD2 was influenced by the artificially integrated *TRA* LCR and monitored in different cell types. Based on this reporter system, the authors detected decreased LCR activity in peripheral mouse T cells and suggested separate gene regulatory mechanisms to be influencing *TRA* expression in mature T cells (11). Later, this regulatory function was attributed to an active element in the 3.9-kb region 5’ of the *TRAC* exons, using a similar mouse reporter system (12). However, so far it could not be pin pointed to any specific mechanisms or element to fulfill this function. Conversely, Eα appeared to be active exclusively during the developmental stage and inactive in mature peripheral T cells (11–15). Only recently, a study found that the deletion of a 1 kb fragment around Eα in a human T cell leukemia cell line led to loss of TCR expression (16).

As the TCR also stands as a pivotal therapeutic target, especially within allogeneic Chimeric Antigen Receptor T (CAR-T) cell therapies, our aim was to conduct a systematic exploration of the *TRA* locus to validate existing or identify novel enhancer elements responsible for regulating rearranged *TRA* expression in mature human T cells (17). Utilizing a pooled and tiled CRISPR screen targeting the *TRAC* gene, along with its 5’ regulatory region and LCR, we aimed to precisely pinpoint essential motifs within enhancers whose disruption results in significant depletion of TCR expression. Identified hits were then validated in human primary T cells.

## 2 Method

### 2.1 Cell culture

Jurkat E6.1 wild type cells (kindly provided by Dr. Anne Eugster, CRTD, Dresden) were cultured in RPMI (Gibco) with 10% fetal bovine serium (FBS) and 1% Penicillin/Streptomycin. For Jurkat TRAC-GFP reporter cells, 10 μg/ml Blasticidin was added. When the reporter cells were subjected to TCR-depletion experiments, antibiotic usage was omitted. Primary T cells were cultured in TexMACS supplemented with IL-2, IL-7, IL-15.

### 2.2 Isolation of human primary T cells

Primary T cells were isolated from peripheral blood mononuclear cells (PBMCs) obtained from buffy coats of healthy blood donors provided by the German Red Cross (Dresden, Germany) after written consent of the donors as previously described (18). The research with human T cells was approved by the local ethics committee of the Medical Faculty Carl Gustav Carus, Technical University Dresden (EK138042014).

### 2.3 Flow cytometry

Cells were stained with anti-human TCRαβ-APC Monoclonal Antibody (IP26) and optionally CD3-FITC Monoclonal antibody (UCHT1) (Thermo Fisher Scientific, Waltham, USA) using MACSQuant X (Miltenyi Biotec, Bergisch Gladbach, Germany) 4 days post electroporation. All samples were analyzed using FlowJo (Becton Dickinson (BD), Franklin Lakes, USA).

### 2.4 mRNA production

CRISPRi mRNA was produced using primers oLi_596 and oLi_921 (**Supplementary Table 7**) and the HiScribe T7 ARCA mRNA Kit with tailing (NEB, Ipswich, England) with partial 5mCTP and Pseudo-UTP (TriLink BioTechnologies, San Diego, USA), according to the manufacturer’s instructions.

### 2.5 Electroporation

Jurkat and primary human T-cells were electroporated using the SE Cell Line Kit or P3 Primary Kit in 20 μl strip format and the Nucleofector 4D (programs “CK-116”, “DQ-115”) according to the manufacturer’s instructions, respectively (Lonza, Basel, Switzerland).

### 2.6 Jurkat TRAC-GFP cell line generation

The donor template was Golden Gate assembled, PCR-amplified and 400 ng electroporated together with 1 pmol Cas9 mRNA (TriLink BioTechnologies) and 10 pmol chemically synthesized sgTRAC-T4 (Synthego, Redwood City, USA) in Jurkat E6.1 cells (**Supplementary Table 6**). After two weeks of Blasticidin selection, single, GFP positive clones were sorted, using a FACSAria Cell Sorter (BD). 53 Single Clones were genotyped for insertion and phenotyped for GFP expression and the highest expressing clone #42 chosen for downstream experiments.

### 2.7 RT-qPCR

RNA was prepared 6 days after electroporation from 5x10^5^ cells using the NucleoSpin RNA Kit (Malcherey-Nagel, Düren, Germany), converted to cDNA using the PrimeScript RT Reagent Kit (Takara Bio, Shiga, Japan) and qPCR performed using Luna^®^ Universal qPCR Master Mix (NEB) in a CFX96™ Real-Time PCR Detection System (Bio Rad, Hercules, USA), all according to the manufacturer’s protocol. A detailed protocol can be found in **Supplementary Table 5**.

### 2.8 sgRNA library cloning and lentiviral production

A detailed protocol can be found in (**Supplementary Table 1**). Briefly, the sgRNA Library was synthesized as an oligo pool (Twist Biosciences), PCR-amplified and inserted into the Lentiviral vector LRT2B expressing TdTomato by Golden Gate cloning (**Supplementary Table 2**) (19). Lentiviral particle production and infection were performed as previously described (20).

### 2.9 Pooled *TRA*-targeted CRISPR screen

Jurkat TRAC-GFP reporter cells were infected at a MOI of 0.1 in triplicates with a 200-fold coverage of the sgRNA library (n = 1908). The infected cells were sorted for TdTomato expression using FACSAria Cell Sorter (BD) and expanded under blasticidin selection. After 3 weeks, 4x10^5^ Jurkat TRAC-GFP cells of each triplicate were electroporated with 1 pmol Cas9 mRNA and 4x10^5^ Jurkat TRAC-GFP cells of each triplicate where additionally cultured without treatment as a baseline control for the sgRNA library. The Cas9-edited cells were stained and the TCRαβ-, GFP- population sorted and expanded. Thereafter, genomic DNA was extracted for the Cas9-edited and non-edited triplicates using the QiAmp DNA Blood Mini Kit (Qiagen, Hilden, Germany), the sgRNA sequences amplified and submitted for 250-bp paired end Illumina deep sequencing (Novogene, Cambridge, UK) (**Supplementary Tables 3** and **4**). The raw reads were pre-processed with Cutadapt v4.7, sgRNA counts per sample were normalized and the median between the replicates and the log2 fold enrichment calculated. Using a one-sided T-test, the p-values for the enrichment of each sgRNA candidate were determined (**Supplementary File** sgTRA_Lib.csv).

### 2.10 Allele-specific genomic analysis

The region around the sgRNA target sites was PCR-amplified from genomic DNA of CRISPR-edited Jurkat clones and subcloned by Golden Gate Assembly. Individual colonies were analyzed by Sanger Sequencing (Microsynth, Balgach, Switzerland).

## 3 Results

### 3.1 Construction of a Jurkat TRAC-GFP reporter cell line

**Figure 1:**
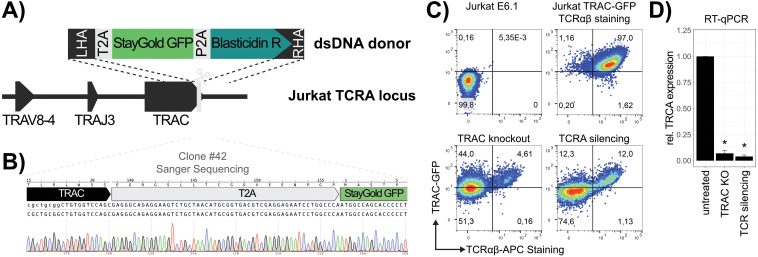
Construction of a Jurkat TRAC-GFP reporter cell line. **(A)** CRISPR knockin strategy of the T2A-StayGold GFP-P2A-BlasticidinR reporter cassette. Cas9-induced double stranded break (DSB) is indicated by a scissor and HDR-mediated integration by dotted lines. **(B)** Validation of correct TRAC-targeting. The Sanger sequencing read of the integrated cassette, amplified by PCR from genomic DNA of Clone #42, is shown. **(C)** Validation of the Jurkat #42 TRAC-GFP cell line by flow cytometry. TCRαβ surface expression was detected by APC-conjugated anti-human antibody and TRAC reporter expression quantified by GFP signal. Cells are represented as pseudocolor dot plots and the percentage of positive cells in each quadrant is indicated for each of the investigated samples. Untreated wild type Jurkat E6.1 were used as a double negative control and untreated TRAC-GFP reporter cells as double positive control. Change in TCRαβ and GFP signal was measured as a consequence of CRISPR/Cas9 knockout (TRAC knockout) or transcriptional silencing (TRA silencing), respectively. **(D)** RT-qPCR of TRAC transcript in untreated and edited samples. Fold change (relative expression) was calculated as 2^-(ΔΔCt)^ to the untreated sample and GAPDH was used as a reference. Bar plots represent the mean fold change from biological duplicates (n = 2) and technical triplicates and their standard deviation is depicted as error bars (p-values *<0.5).

In order to provide a straightforward and reliable method for assessing the transcriptional state of the *TRAC* gene, a Jurkat TRAC-GFP reporter cell line was constructed. At first, the identity of the rearranged *TRA* locus was resolved using publicly available RNA-seq data and revealed that Jurkat E6.1 cells express a TCRα chain consisting of *TRAV8-4, TRAJ3 and TRAC* (**Figure 1A**). A dsDNA fragment containing a *T2A-StayGold GFP-P2A-BlasticidinR* cassette was inserted in-frame downstream of the final *TRAC* exon by homology directed repair (HDR)-mediated CRISPR/Cas9 knock-in (**Figure 1A**). The edited Jurkat E6.1 cells were selected in blasticidin, sorted for GFP expression and individual clones were analyzed by flow cytometry, PCR and Sanger sequencing. Clone #42 was confirmed to have inserted the GFP cassette as intended and expressed GFP ubiquitously (**Figure 1B**; **Supplementary Figure 1**). It was therefore designated for subsequent experiments (Jurkat TRAC-GFP).

To validate the functionality of the cell line as a reporter for *TRAC* expression, we performed TCR knockout and silencing experiments (**Figure 1C**). The change in GFP expression was investigated as a consequence of TCR knockout, simulating the disruption of *TRAC* exons and transcriptional silencing, simulating the loss of an enhancer element, respectively. These mechanisms represent the two main events that are expected to be observed in the pooled CRISPR/Cas9 screen (**Supplementary Figure 2**). The knockout was performed using a *TRAC* targeting sgRNA (sgTRAC-1; **Supplementary Table 6**), in combination with Cas9 mRNA. Co-electroporation of both reagents resulted in almost complete loss (95%) of detectable surface expression of TCRαβ, but around 50% of the cell pool maintained functional transcription of the downstream GFP reporter (**Figure 1C**). Because the mechanism of transcriptional silencing acts directly on the promoter, this method is more suitable to simulate the transcriptional regulation of the *TRA* by enhancer elements. It thereby prevents the expression of both, TCRα and GFP. To test the effect of transcriptional silencing, the promoter of the *TRA* gene in Jurkat TRAC-GFP cells, which was identified to be upstream of *TRAV8-4*, was targeted using sgTRA-1 (**Supplementary Table 6**) in combination with mRNA of a CRISPR interference (CRISPRi, 21). The transcriptional silencing impaired the TCRαβ surface expression in 85% of the cells and also decreased the GFP signal in 74% of cells. To further validate this result, RT-qPCR was performed, which indicated the efficient depletion of *TRA* transcripts (**Figure 1D**). Taken together, these data demonstrate that the Jurkat TRAC-GFP cell line is suitable as a reporter for the dissection of regulatory elements that govern the expression of the *TRA* locus. This tool enabled us to genetically investigate the effects of regulatory elements on the expression of TCRα on RNA and consequently also on protein level.

### 3.2 TRA pooled CRISPR/Cas9 screen

**Figure 2:**
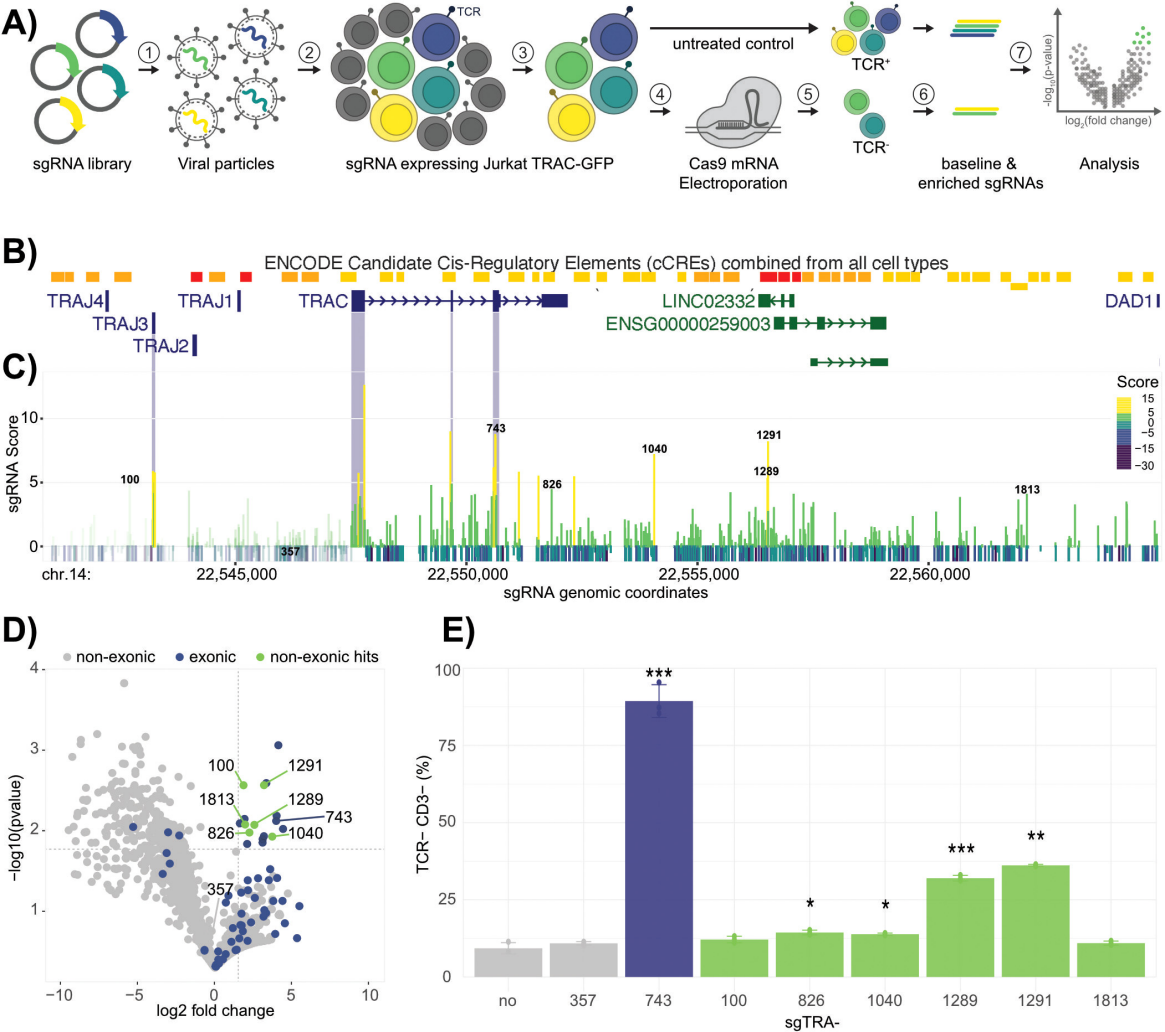
TRA pooled sgRNA Library Screen. **(A)** Workflow of the pooled sgRNA library screen. (1) lentiviral particles harboring the sgRNA library and a TdTomato marker. (2) Jurkat TRAC-GFP cell transduction at a MOI of 0.1 followed by (3) sorting into a pure sgRNA-expressing population, based on the TdTomato marker. (4) Electroporation of sgRNA expressing cell pool with Cas9 mRNA followed by (5) sorting for GFP- and TCRαβ- cells. (6) sgRNA was amplified from genomic DNA of the sorted and untreated control samples followed by (7) analyzes by deep sequencing. **(B)** UCSC genome browser view of the targeted region. Annotated transcripts and cis-regulatory elements are indicated. **(C)** Screen results. Genomic coordinates of each sgRNA and their calculated score (-log10(pvalue)*log2(fold change)) are shown by an indicated color code. The most relevant sgRNAs are labeled with their IDs. sgRNAs targeting TRAC exons and the TRAJ3 region are highlighted by pale blue bars. In the applied Jurkat cell line, the TRA locus is rearranged, differing from the genome browser view. Therefore, the regions that are excised in the Jurkat genome are indicated by pale color of the bar, since the respective sgRNAs are not expected to bind. **(D)** Volcano plot of non-exonic (grey), exonic (blue) and enriched non-exonic (green) sgRNAs. Cut-offs for the classification of enriched sgRNAs are depicted by grey, dotted lines. **(E)** Validation of individual sgRNA hits in Jurkat E6.1. Cells were Cas9-edited with the respective sgRNA candidate (25 pmol) and stained for TCRαβ and CD3 (p-values compared to no-sgRNA sample: ***< 0.001, **< 0.1, *<0.5). Colors represent the classification from **(D)**.

To screen for putative genetic elements that influence TCRα expression in Jurkat TRAC-GFP cells, a CRISPR/Cas9-mediated approach was chosen over CRISPRi. While CRISPRi can robustly silences promoters, its impact on enhancer elements is highly variable, influenced by chromatin context, transcription factor susceptibility, and the overall regulatory network (22). In contrast, the CRISPR/Cas9 nuclease approach creates permanent sequence disruptions, enabling to precisely probe the effects of both exonic knockout and non-coding region deletions on TCRα expression in its native regulatory environment. A sgRNA library was designed *in silico*, targeting every possible NGG protospacer adjacent motif (PAM) from around 6.5 kb upstream of *TRAC* (chr.14:22,541,009 in hg38) to the next downstream gene *DAD1* (chr.14: 22,564,966 in hg38). This analysis resulted in a total of 1906 sgRNAs. The region was chosen in order to include any possible regulatory element directly upstream of *TRAC* and within the downstream intergenic region, where several enhancer elements had been annotated (5, 6). Furthermore, intronic regions were investigated with exonic regions serving as positive controls, because their disruption would likely result in a gene knockout and enrichment of those sgRNAs in the screen. First, the sgTRA library was inserted downstream of a U6 promoter into the lentiviral vector LRT2B, harboring TdTomato (19). Jurkat TRAC-GFP reporter cells were then infected at a multiplicity of infection (MOI) of 0.1 to ensure that most cells integrated only a single sgRNA cassette. The infection was performed in triplicates and the cells were sorted into a pure, sgRNA-expressing population, using the TdTomato marker (**Figure 2A**). Each triplicate culture was divided into two samples. In the first sample, no treatment was applied, preserving the baseline distribution of the sgRNA library as a control. The second sample was electroporated with Cas9 mRNA, enabling effective genome editing (**Figure 2A**). Following electroporation, double negative GFP-/TCRαβ- cells were isolated by fluorescence activated cell sorting (FACS), as this population reflects the subset in which *TRA* expression was successfully disrupted by Cas9 editing. These GFP-/TCRαβ- cells were then expanded to ensure sufficient material for downstream analysis. To identify the specific sgRNAs present in the TCRαβ- cell population, genomic DNA was extracted from each sample, and sgRNA sequences were PCR-amplified. This amplification was performed on both the Cas9-edited, TCRαβ- cells and on the untreated baseline samples, allowing for direct comparison. Following PCR, the sgRNA counts were quantified by Ilumina 250-bp paired-end deep sequencing, providing a high-resolution profile of sgRNA representation in each condition. The sgRNAs were scored based on the significance of their enrichment across replicates (p-value) and the magnitude of enrichment (log fold change). The reads were then mapped to their genomic coordinates to visualize clustering of enriched sgRNAs (**Figure 2B**). As expected, many (79%) of the exon-targeting sgRNAs scored highly with enrichment peaks directly mapping to the corresponding TRAC exons (**Figure 2C**). Moreover, multiple enriched sgRNAs targeting the exons of TRAJ3, but not TRAJ1, TRAJ2 or TRAJ4 were identified in the GFP- fraction, indicating that sequence alterations in TRAJ3 influence the expression of the TRAC locus (**Supplementary Figure 3**). This observation is in line with the fact that, determined from RNA-seq data, the Jurkat TCRα chain is expressed from the rearranged TRAJ3 segment (23). Collectively, this data demonstrates the suitability of the assay to uncover genomic regions important to maintain TRAC expression.

Within the volcano plot cut-off thresholds, (LFC > 1.5; p-value < 0.02) six non-exonic hits were identified, namely sgTRA-100; 826; 1040; 1289; 1291 and sgTRA-1813 (**Figure 2D**). Of particular interest were sgTRA-100, as well as sgTRA-1289 and 1291, binding in Eα element Tα2.

### 3.3 Independent validation of individual sgRNA hits

**Figure 3:**
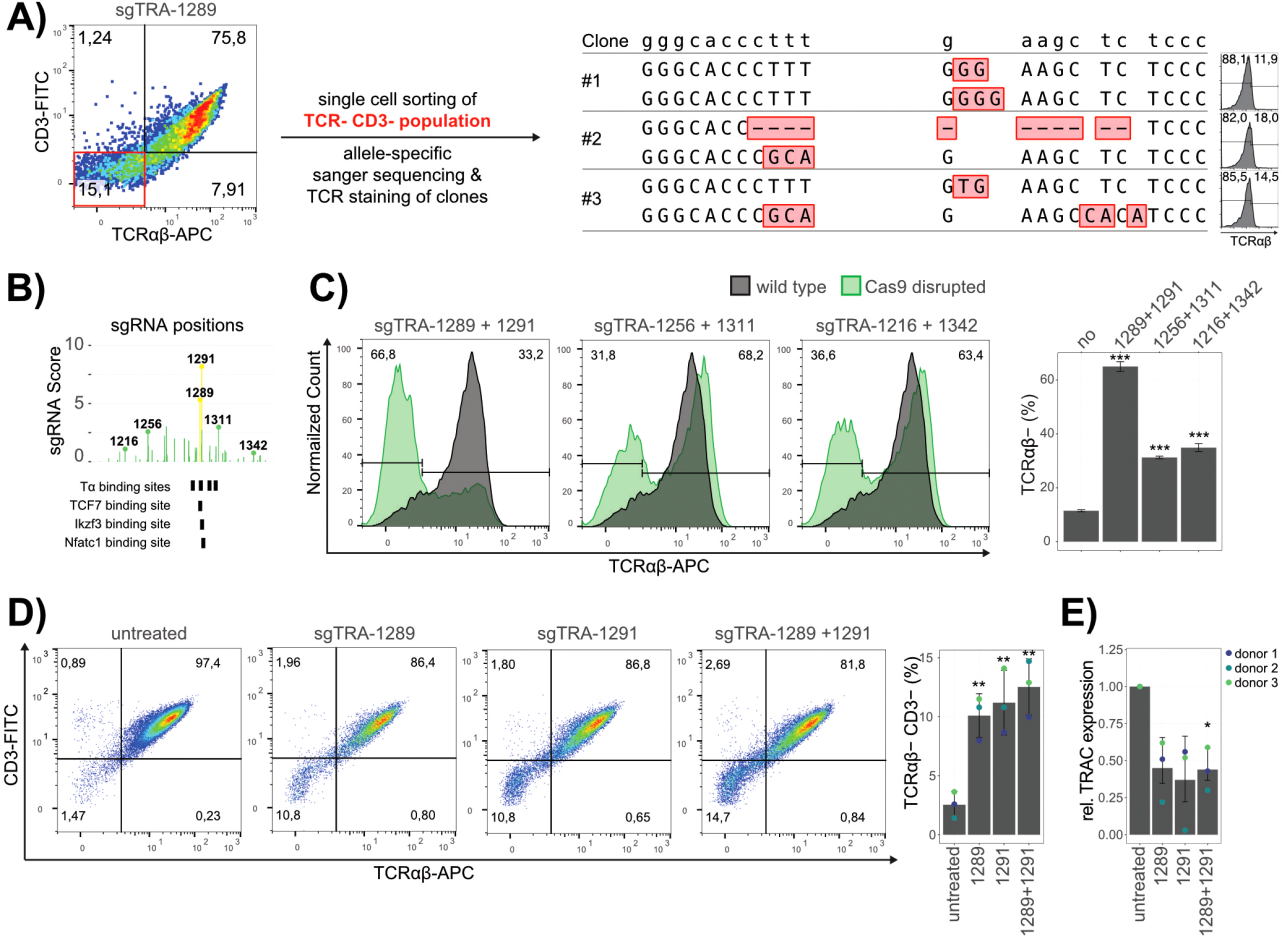
Validation of sgRNA Hits. **(A)** Genomic analysis of TCRαβ- Jurkat TRAC-GFP clones after editing. Double CD3 and TCRαβ negative cells (red quadrant in the left FACS panel) were single cell sorted and their allele-specific genomic sequence, as well as their surface TCRαβ expression analyzed. The reference genomic sequence is shown at the top and the sequence of the two alleles of each clone is depicted below with Indels marked in red. Flow cytometry histograms to the right indicate the surface expression of TCRαβ for the corresponding clones. **(B)** Genomic region of Eα with mapped sgRNAs and their scores from the screen. Bold numbers and lollipop bars indicate the sgRNAs used for excision of different fragment sizes spanning around Eα. Below, Tα and relevant transcription factors binding sites are indicated. **(C)** Representative flow cytometry analysis of cells edited with Cas9 and the indicated sgRNAs, represented as overlay with unedited, TCRαβ stained cells (grey). The percentages of TCRαβ- cells are indicated. Data of three replicates is quantified in a barplot (right panel). **(D)** Representative flow cytometry analyses of primary T cells edited with Cas9 and sgRNA hits (50 pmol), as well as a combination of sgTRA-1289 and sgTRA-1291 (25 pmol each) is shown to the left with percentages for each quadrant indicated. The data of cells from three different donors is quantified in the bar plot to the right, with error bars representing the standard deviation between donors (n=3). **(E)** TRAC expression analysis. RT-qPCR results of TRAC performed in technical triplicates for the cells from three donors is shown. GAPDH was used as a reference and the fold change (relative expression) calculated as 2^-(ΔΔCt)^ is presented. Dots represent the mean fold change and error bars the standard deviation between donors (n=3). Statistical significance was determined by students t-test. P-values compared to untreated samples are represented as stars in bar plots (***< 0.001, **< 0.1, *<0.5).

In order to validate the obtained hits, the sgRNAs of interest where chemically synthesized and electroporated individually into Jurkat E6.1 wild type cells together with Cas9 mRNA. As controls, the enriched exonic hit (sgTRA-743) was used as a positive control, whereas a non-enriched, non-exonic sgRNA (sgTRA-357) was chosen as negative control. To allow for a precise assessment of the influence on *TRA* expression, the TCR complex was antibody stained for both TCRαβ and CD3 in these experiments. The samples treated with sgTRA-100 revealed no significant influence on *TRA* expression, meaning that no relevant regulatory role of the 5’ J region could be validated. A slight interference with *TRA* expression was observed for sgTRA-826 and sgTRA-1040, reflected by modest reductions of 5% and 4.5%, respectively, in the TCRαβ^+^ CD3^+^ Jurkat cell population. These findings suggest that these regions contribute mildly to the transcriptional regulation of *TRA*. In contrast, the hits for sgTRA-1289 and sgTRA-1291 could be validated with strong confidence (**Figure 2E**; **Supplementary Figure 4**). The perturbation with these sgRNAs led to a significant reduction of 23% and 27%, respectively, though this reduction was more moderate compared to the robust effect observed with the exon-targeting positive control sgTRA-743 (89%). To further validate that the reduction in *TRA* expression indeed resulted from the sequence disruption of Tα2, the subset of TCRαβ- CD3- cells, obtained by the validation experiment of sgTRA-1289 were sorted to single clones. Three independent clones were picked, analyzed for TCRαβ by flow cytometry, their genomic DNA isolated and the target site for sgTRA-1289 amplified and subcloned for sanger sequencing at single allele resolution (**Figure 3A**). The three clones were all negative for TCRαβ and showed indels on both alleles, further validating that loss of *TRA* expression can be directly linked to the disruption of Tα2.

In order to intensify the effect of enhancer disruption on *TRA* expression, cells were treated with two sgRNAs, which could result in the frequent excision of the intermediate DNA piece and loss of functional enhancer elements. Different sgRNA combinations resulting in the excision of different sizes of enhancer DNA fragments were tested (sgTRA-1289 + sgTRA-1291: 25bp; sgTRA-1256 + sgTRA-1311: 397bp; sgTRA-1216 + sgTRA-1342: 985bp) (**Figure 3B**). These results indicated that treatment with the two sgRNA closest to the enhancer element Tα2 (sgTRA-1289 + sgTRA-1291) impaired the TCRα expression in a major population (66.8%) of the cells (**Figure 3C**). Treatment with sgRNAs more distant from Tα2 also resulted in impaired TCRα expression, but at a reduced efficiency (31.8%; 36.6%). This suggests that the proximal region outside the sgTRA-1289 and sgTRA-1291 editing window lacks synergistic enhancer activity capable of significantly influencing TRA expression, emphasizing the central role of the Tα2 sequence in *TRA* regulation. Alternatively, this effect might be impacted by less efficient editing to achieve larger deletions. Altogether, these results identify Tα2 as an important regulatory element to maintain *TRA* expression in Jurkat cells.

To determine whether these effects are specific to Jurkat cells or are also applicable to primary human T cells, we extended our analysis to mature T cells from peripheral blood. CD3+ cells were isolated from three healthy donors und subjected to editing with Cas9 mRNA and the most effective sgRNAs (sgTRA-1289, sgTRA-1291) (**Figure 3D**; **Supplementary Figure 5**). After editing, although lower than in Jurkat, a clear population of TCRαβ and CD3-depleted cells could be observed and was more pronounced when both sgRNAs were combined (**Figure 3D**). To prove that the low percentage of cells in the population did not result from ineffective Cas9 cutting, sanger sequencing of the genomic DNA was performed and quantified with an Inference of CRISPR Edits (ICE) analysis. This analysis validated ~ 90% efficient cleavage in the samples (**Supplementary Figure 6**) (24). Furthermore, sequencing of the TCRαβ^−^/CD3^−^ subpopulation revealed a specific deletion of the Tα2 element in all investigated clones, reinforcing the link between Tα2 disruption and TRA downregulation (**Supplementary Figure 7**). Notably, the milder phenotype observed in primary T cells may be influenced by the heterogeneity of the cell population or differences in DNA repair mechanisms between Jurkat and primary T cells following CRISPR/Cas9 editing. Using RT-qPCR, we furthermore verified that the reduced TCR surface expression also correlated with a reduced abundance of the *TRAC* transcript in the edited cells (**Figure 3E**). These results confirm that the observed effects of Tα2 disruption on TCR expression extend beyond the Jurkat cell model and are also applicable to primary human T cells. Altogether, this reinforces the relevance of Eα in regulating *TRA* expression, specifically through the Tα2 element in peripheral T-cells in a physiological, human-derived context.

## 4 Discussion

Understanding the regulation of the *TRA* gene is fundamental to advancing our knowledge of T cell biology. The TCR which is crucial for T cell activation and immune response, is tightly regulated, with *TRA* expression playing a central role in proper T cell development and function (25, 26). A clearer understanding of these mechanisms also paves the way for improved therapeutic strategies, such as CAR-T cell therapies, where precise control of TCR expression can enhance both safety and efficacy (27). This knowledge is particularly critical in allogeneic settings, where modulating TCR expression can prevent adverse immune responses and optimize therapeutic outcomes (17). Previous studies investigating *TRA* regulation have often employed mouse models, artificial reporter constructs or cell lines to draw conclusions about the regulatory functions of elements like Eα in mature peripheral T cells (12, 14, 16). While these models provided valuable initial insights, they may not fully capture the complexities of *TRA* regulation in a native human cellular context.

By exploiting the power of a targeted pooled CRISPR screen, we probed 24 kb of the *TRA* locus for the existence of functional elements whose disruption would lead to an impaired TCR expression in mature human T cells. Hence, this work systematically screened for the existence and functionality of small deletions leading to altered *TRA* expression in the native genetic context of human peripheral T cells. Together, the obtained data could help to confirm or challenge current hypothesis about *TRA* regulation.

Previous data suggested the existence of a regulatory element in the 3.9 kb region upstream of the *TRAC* exons, which might be driving TCRα expression (12). However, this finding has recently been challenged (16). Among the 490 sgRNAs covering this region at a high density, only sgTRA-100 was standing out as an enriched hit. However, its TCR-depleting effect could not be validated in an independent experiment. Therefore, the existence of a regulatory element in the 3’-Jα region whose disruption would lead to impaired *TRA* expression could not be confirmed in the human Jurkat model cell line. On the other hand, Eα was thought to be inactive in mature T cells (11, 14, 15), even though this view was recently challenged by Rodríguez-Caparrós et al. (16). Previous experiments investigated a transgenic mouse model with an integrated *TRA* LCR that drove the expression of hCD2 and showed reduced LCR activity in peripheral T cells (11). Another study pointed to reduced transcript levels, associated with Eα in peripheral T cells of transgenic mouse models and concluded that Eα is inactivated in αβ T lymphocytes (14). Only Rodríguez-Caparrós et al. recently demonstrated that the deletion of a 1 kb fragment around Eα in Jurkat cells was required to abrogate TCRα expression.

The data provided in this work demonstrates that in human peripheral T cells the disruption of Eα indeed leads to a decrease in *TRA* expression on the transcriptional and protein level and pinpoints this effect to the Tα2 element. Future research should explore whether these discrepancies to earlier work arise from species-specific differences in T cell regulation or if they point to a broader, more fundamental mechanism underlying *TRA* expression across species.

Two independent sgRNAs, sgTRA-1289 and sgTRA-1291 targeting Tα2, were enriched in the screen and could be validated in subsequent experiments. Furthermore, Jurkat E6.1 clones with sgTRA-1289-edited Tα2 sequence showed almost complete loss of TCR expression. Interestingly, the sgTRA-1289 cleavage site specifically overlaps with a binding site for the transcription factor TCF7/Lymphoid enhancer-binding factor-1 (TCF7/Lef1) 5’-CTTTGA-3’, which is also part of the TRA enhanceosome (**Figure 3B**) (13, 28). In close proximity, sgTRA-1291 is also directly interfering with the binding sites for Ikaros family zinc finger 3 (Ikzf3) and nuclear factor of activated T cells, cytoplasmic 1 (Nfatc1). These factors are involved in regulating T cell activation (29). Although the detailed regulatory mechanism driving *TRA* expression in mature lymphocytes is not yet clear, the provided data demonstrates that the disrupting of Tα2 by two independent sgRNAs resulted in the loss of *TRA* transcription and surface expression in a subset of human, mature, peripheral, primary T lymphocytes. Future work could investigate if this subset is phenotypically distinct and how disruption of this element influences the binding of transcription factors and chromatin organization to provide further detail on transcriptional regulation of *TRA*.

While this employed screen successfully identified essential enhancer elements in Jurkat cells, certain limitations should be acknowledged. First, our work focused on cis-regulatory elements within a defined enhancer region, and distal or trans-regulatory elements were not assessed. Second, although Jurkat cells are widely used as a model for T-cell biology, their regulatory mechanisms may differ from those in primary T cells. Primary T cells provide a more physiologically relevant model, yet pooled CRISPR screens in these cells are constrained by technical challenges such as lower transduction efficiency, as well as limited culture time and viability. Moreover, while on-target sanger sequencing revealed specific deletions of the Tα2 element, we cannot entirely rule out the possibility that Cas9-mediated editing resulted also in large deletions affecting the *TRAC* coding sequence in a small subset of T cells, which in rare cases could contribute to the observed loss of *TCRα* expression.

Taken together, this work suggests that Eα is active in mature peripheral human T cells and required for *TRA* expression. Moreover, TCRα expression could be abrogated only by the specific disruption of Tα2 in a subset of T cells, indicating its essential role in Eα function. This finding not only advances our understanding of T cell receptor regulation but also hold implications for CAR T cell therapies, where precise control of TCR expression is essential. Insights into Eα‘s activity could support the development of strategies to modulate TCR expression more effectively in allogeneic contexts. Towards this goal, further work is necessary to understand the detailed interaction of regulatory factors with Tα2 and which specific DNA motifs are necessary for binding and active transcription.

## Data Availability

The original contributions presented in the study are included in the article/**Supplementary Material**. Further inquiries can be directed to the corresponding author.
