# Supplementary material for: A pooled CRISPR screen identifies the Tα2 enhancer element as a driver of TRA expression in a subset of mature human T lymphocytes
Source: Front Immunol. 2025 Mar 14;16:1536003. doi: 10.3389/fimmu.2025.1536003 (PMC11949936; doi:10.3389/fimmu.2025.1536003)
Supplement: Supplementary file 2 [file DataSheet2.docx]

Supplementary Material

# Supplementary Figures


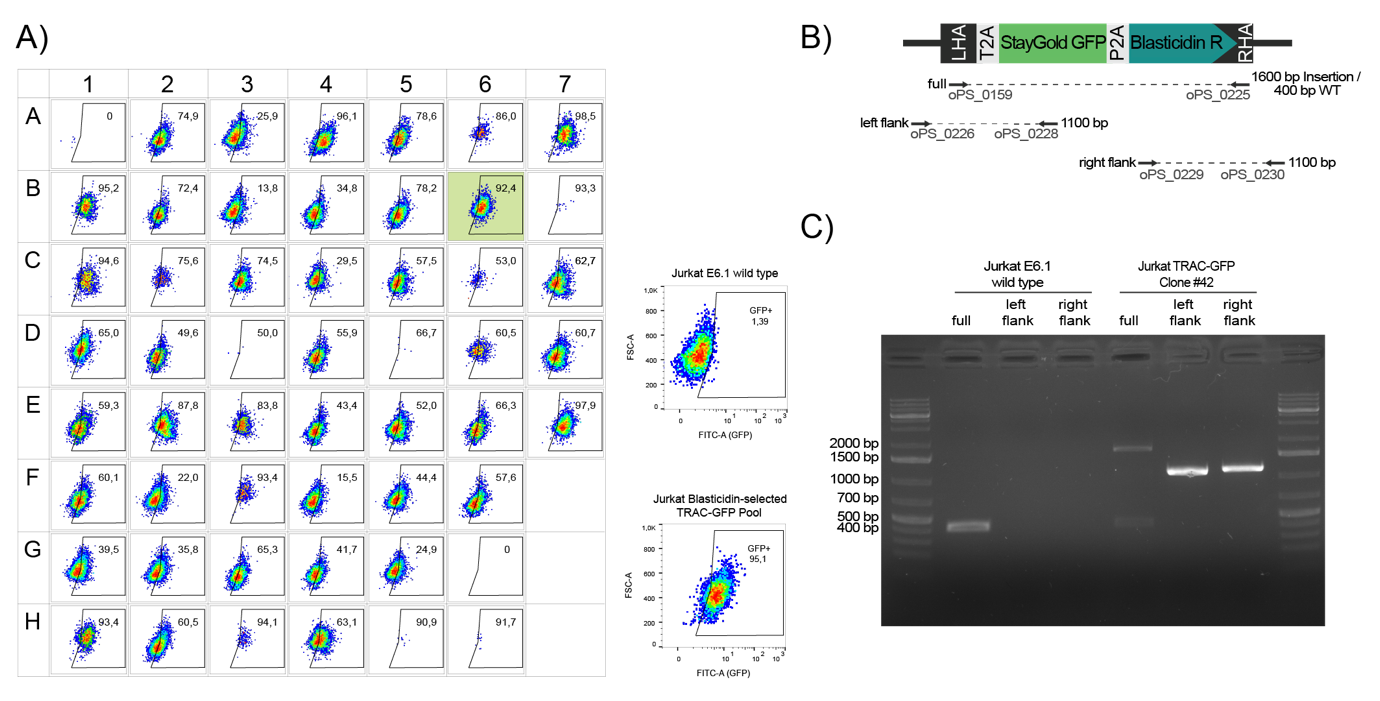


Supplementary Figure 1: Jurkat TRAC-GFP Genotyping and Phenotyping. (A) Phenotyping by flow cytometry for GFP expression of individual Jurkat TRAC-GFP clones. The chosen clone #42 is marked in green. (B) PCR Strategy for genotyping of knocked-in reporter construct. The schematic on the top represents the genomic integration site of the reporter construct with its left and right homology arms (LHA, RHA), T2A-GFP and P2A-Blasticidine resistance cassettes. Arrows indicate primers with their indicated names and dotted lines indicate the respective amplicons. PCRs were performed stretching the left homology arm to the genomic DNA “left flank” and the right homology arm to the genomic DNA “right flank”, as well as over the whole insert “Insertion” (C) PCR products for genotyping of clone #42 and wild type control are visualized on an agarose gel image.


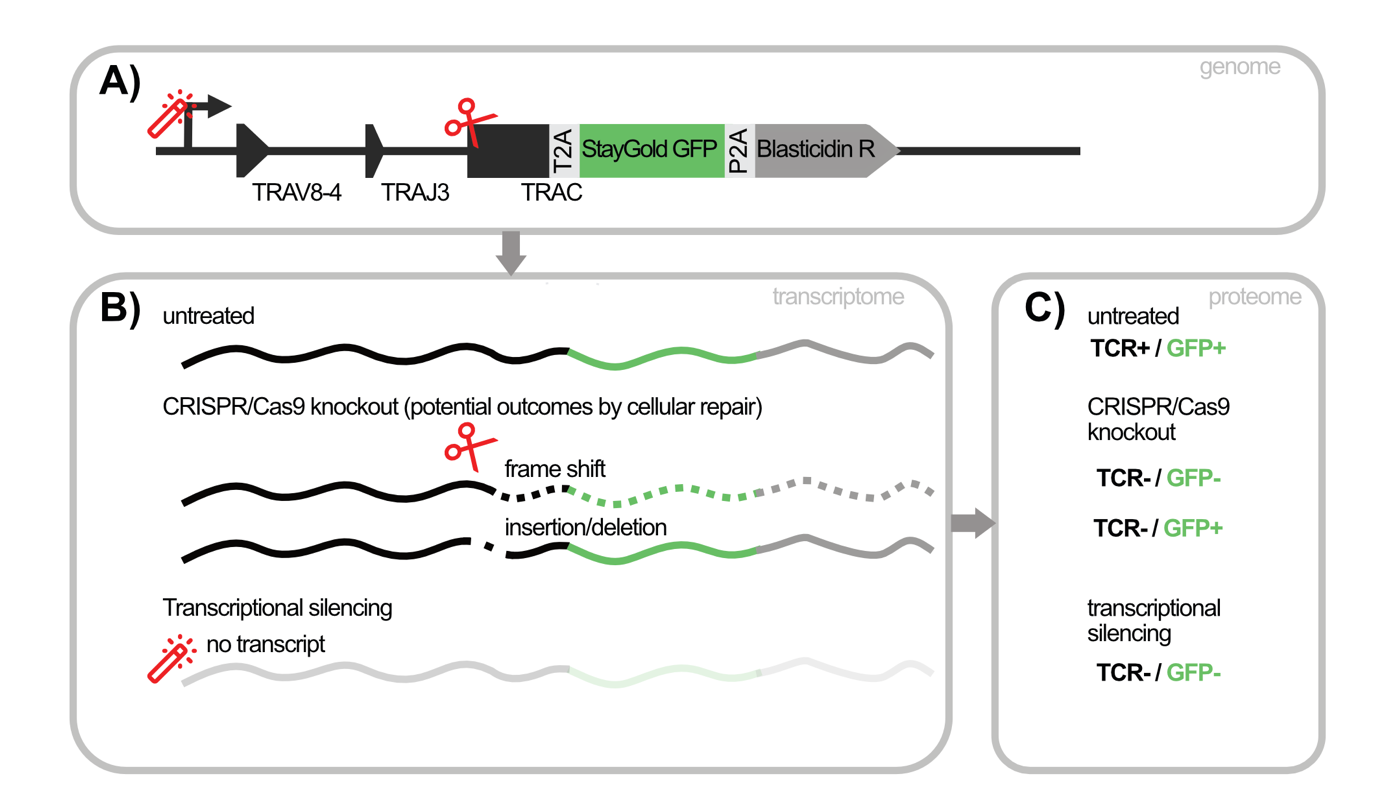


Supplementary Figure 2: Schematic of reporter line functionality in knockout vs. silencing context. (A) The genomic sequence of the TCRA locus and reporter construct in the Jurkat TRAC-GFP reporter cell line is described with the targets for CRISPR/Cas9 knockout (red scissor) and transcriptional silencing (red magic wand). (B) The outcome of both editing techniques on the transcriptional level and subsequently (C) the proteomic level is illustrated. During the cellular repair process induced by CRISPR/Cas9 knockout, indels are introduced into the DNA sequence, often causing frameshifts that disrupt the whole transcript and result in the loss of both TCRA and GFP expression. However, some indels may only lead to the addition, deletion, or substitution of amino acids at the protein level. This can impair folding, surface presentation, and antibody binding of the targeted TCR alpha chain, but would not affect the transcription or expression of the downstream GFP reporter. The transcriptional silencing on the other hand, directly acts on the promoter and prevents the transcription of any mRNA, resulting in loss of both, TCRA and GFP expression.


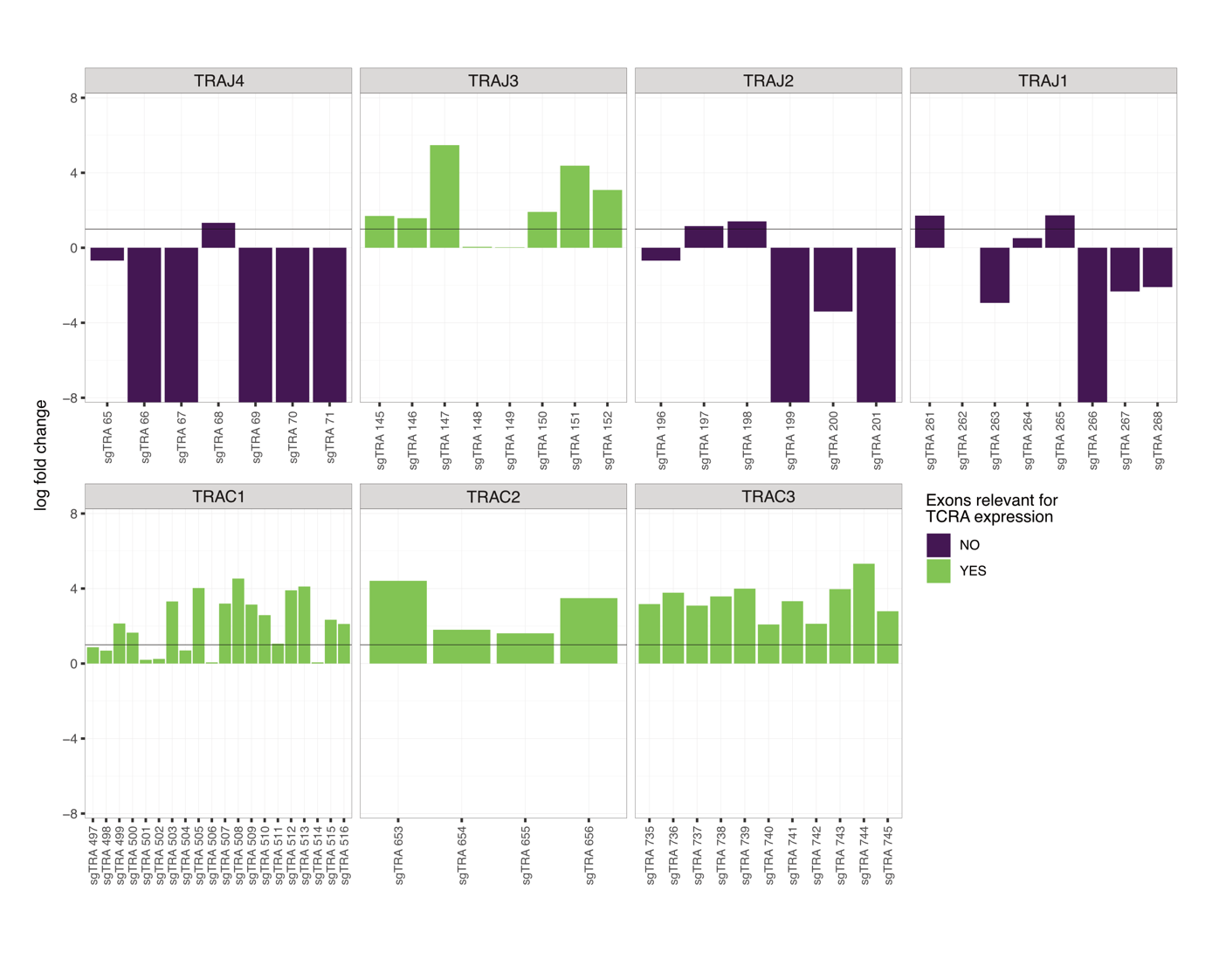


Supplementary Figure 3: Enrichment of exonic sgRNAs as positive controls of the pooled CRISPR/Cas9 screen. Every panel represent one exon and contains the sgRNAs that are targeting this specific exon and their respective fold change after positive selection. sgRNAs targeting exons, which are relevant for TCRA expression are colored in green and most of them were strongly enriched, as expected from the positive control. sgRNAs targeting exons irrelevant for TCRA expression are colored in dark violet and were mostly lost or de-riched in the screen.


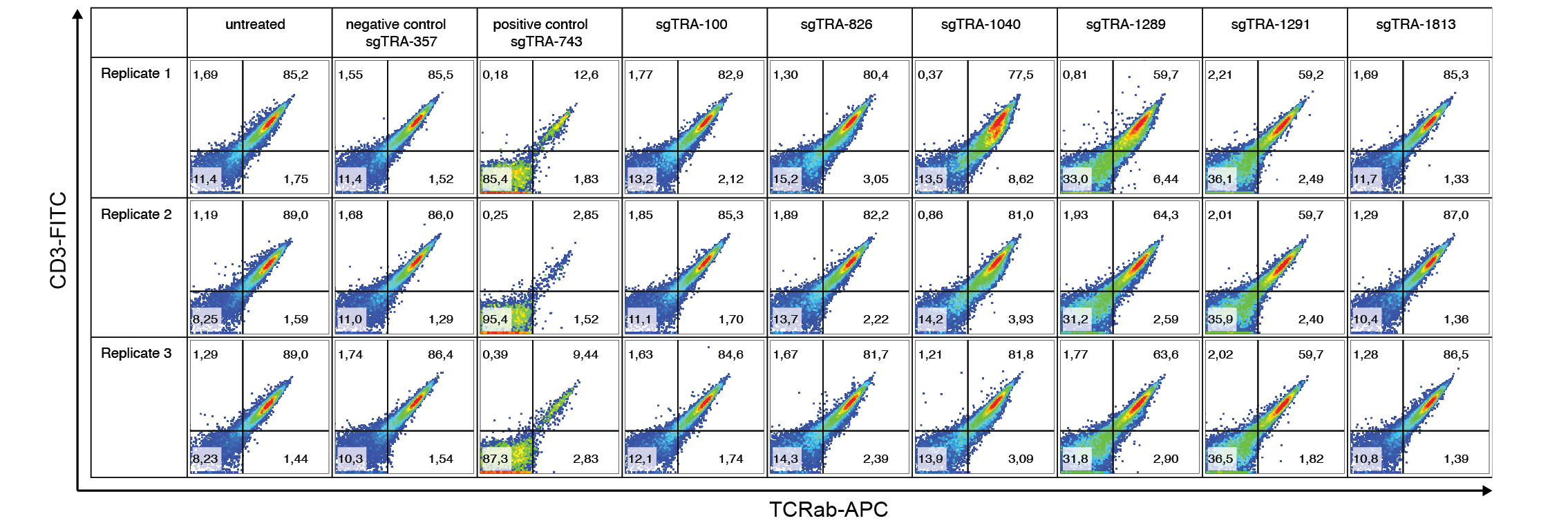


Supplementary Figure 4: Flow Cytometry replicates of independent sgRNA validation in Jurkat. Each column represents the flow cytometry results of Jurkat E6.1 cells, electroporated with Cas9 mRNA and individual chemically synthesized sgRNAs in triplicates. The cells were stained with CD3-FITC and TCRαβ-APC antibodies and the percentage of positive cells in each quadrant is indicated with a number.


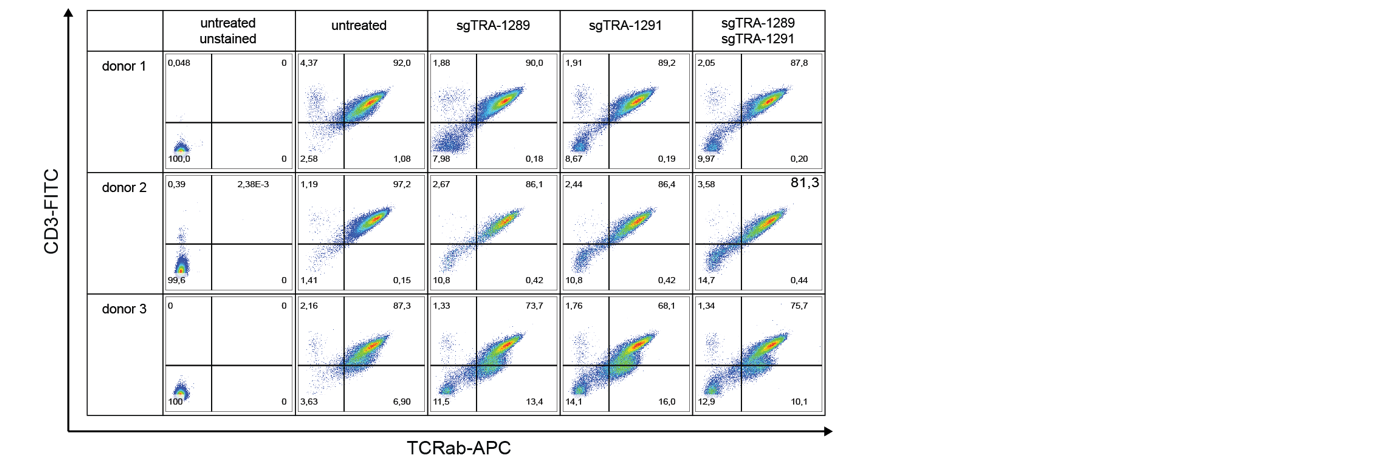


Supplementary Figure 5: Flow Cytometry replicates of primary T cells. Each column represents the flow cytometry results of primary T cells obtained from three different donors, electroporated with Cas9 mRNA and individual chemically synthesized sgRNAs. The cells were stained with CD3-FITC and TCRαβ-APC antibodies and the percentage of positive cells in each quadrant is indicated with a number.


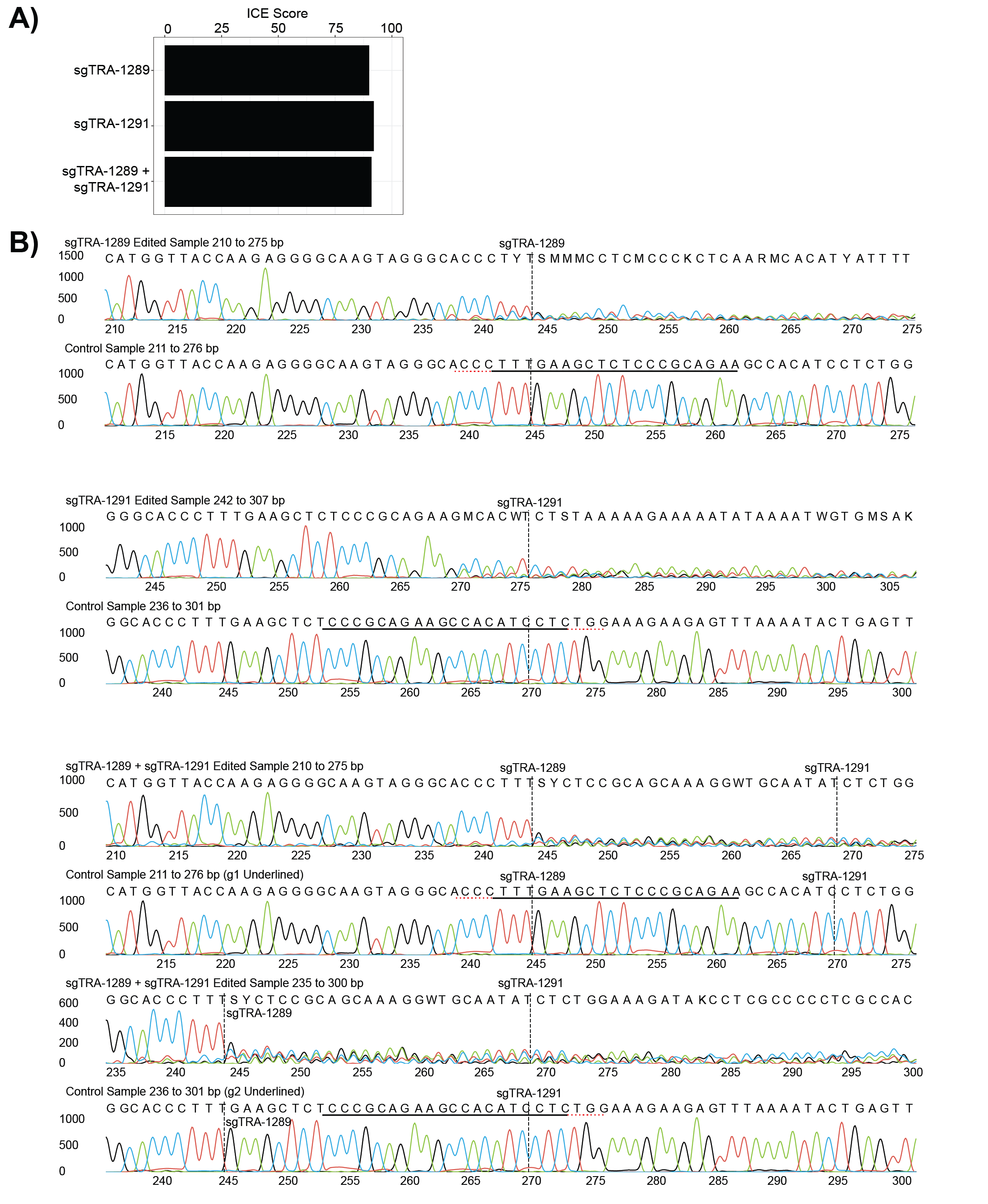


Supplementary Figure 6: Inference of CRISPR Edits (ICE) for representative primary T cell samples. Efficient Cas9 cleavage of enhancer alpha with sgTRA-1289 and sgTRA-1291 was proven by Sanger Sequencing and ICE analysis of the cell pool 6 days post electroporation. (A) Calculated ICE Score is displayed for Cas9 editing with different sgRNAs in representative samples from donor 1. (B) Original Sanger Sequencing reads used for ICE analysis. The Sanger reads of an edited sample, indicated by the sgRNA name and an untreated “Control Sample” are compared and displayed in paires. The spacer sequence is indicated as the underlined DNA sequence in the “Control Sample”, the PAM as red dotted line and the cleavage site as a black vertical dotted line. The pair represents the sanger sequencing histograms from untreated samples compared to samples edited with Cas9 and sgTRA-1289, edited with sgTRA-1291, or edited with sgTRA-1289 and sgTRA-1291, respectively. Lower histogram peaks indicate an increased frequences of indels downstream of the cleavage site, which were used to calculate the respective ICE scores.


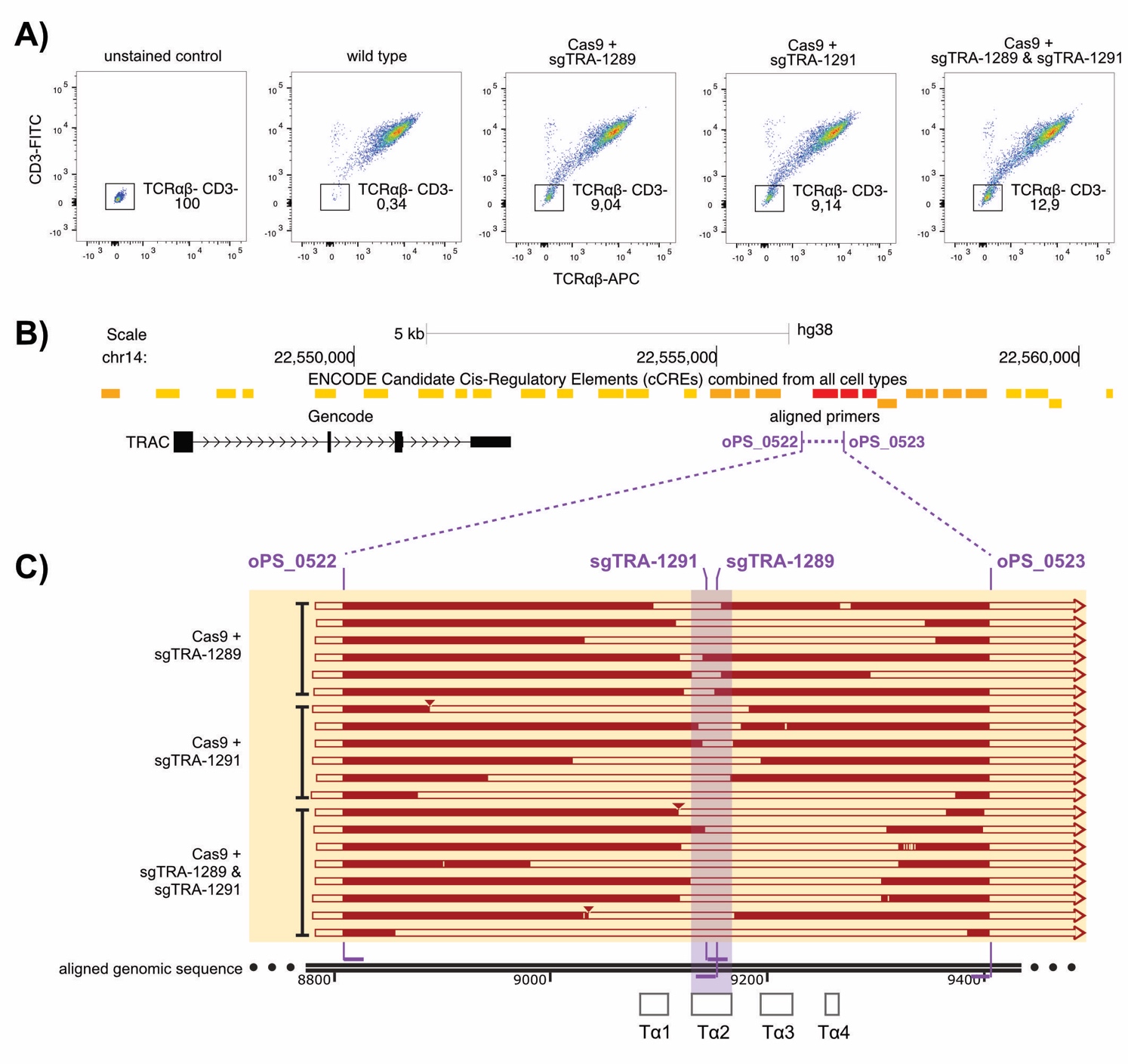
**Supplementary Figure 7: Genomic investigation of TCR-depleted primary T cell subpopulation.** (A) Flow cytometry data of primary, Cas9-edited T cells with sgTRA-1289, sgTRA-1291 or both, respectively at 4 dpt (timepoint of FACS sorting). The gate for TCRαβ^-^/CD3^-^ cells is indicated by a box and the percentage of TCRαβ^-^/CD3^-^ labeled. The pool of TCRαβ^-^/CD3^-^ cells was FACS sorted for each sample and genomic DNA extracted. (B) Genome browser view of the *TRAC* gene and the downstream Eα region. Primers to PCR-amplify the region of interest are aligned and labeled in purple. (C) Zoom into the amplified region with Tα elements indicated by white boxes in the bottom. PCR with the indicated primers was conducted on the genomic DNA, subcloned into a plasmid and individual clones analyzed by Sanger Sequencing. The Sanger reads are indicated as red bars. Filled bars indicate unmodified sequences, while empty bars indicate deletions and arrows indicate insertions. The samples corresponding to the reads are labeled on the left. The Tα2 element is highlighted in purple to pinpoint all deletions affecting the motif.

# Supplementary Methods

## sgRNA library cloning protocol

### Amplification of oligo pool

The lyophilized synthesized oligos (Twist Bioscience) were reconstituted in 20 μl of nuclease-free water, to obtain a concentration of 2 ng/μl and used as template for PCR with the NEB Next Ultra II Q5 Master Mix (NEB), as to see below. From the resulting PCR product, 5 μl were used for quality control on a 1% agarose gel, 45 μl were cleaned up using the Isolate II PCR and Gel Kit (Bioline) and the concentration determined with the NanoPhotometer (Implen).

Supplementary Table 1: Ultra II Q5 Protocol

| **x1 (50 μl)** |  | **Program:** |  |  |
| --- | --- | --- | --- | --- |
| 25 μl | Ultra II Q5 Master Mix | 98°C | 30 sec |  |
| 5 μl | oPS_0273 (10 μM) | 98°C | 10 sec | 20 Cycles |
| 5 μl | oPS_0274 (10 μM) | 59°C | 30 sec |  |
| 10 μl | gRNA Pool (2 ng/μl) | 72°C | 30 sec |  |
| 5 μl | Nuclease-free water | 72°C | 2 min |  |
|  |  | 8°C | Hold |  |

### Golden Gate library cloning

The Golden Gate Assembly for insertion of the gRNA library was performed as to see below:

Supplementary Table 2: Golden Gate Library Cloning Protocol

| **x1 (20 μl)** |  | **Program:** |  |  |
| --- | --- | --- | --- | --- |
| 14 μl | Nuclease-free H2O | 42°C | 10 min | Initial digest |
| 1 μl | LRT2B vector (25 fmol) | 42°C | 3 min | 20 Cycles (digest/ligation) |
| 1 μl | Amplified gRNA Pool (100 fmol) | 16°C | 3 min |  |
| 2 μl | T4 DNA Ligase Buffer (10X) | 42°C | 10 min | Final digest |
| 1 μl | T4 DNA Ligase (NEB) | 60°C | 10 min | Inactivation |
| 1 μl | BsmBI-v2 (NEB) | 8°C | Hold |  |

### Bacterial transformation

The Golden Gate ligation mix was purified over a membrane on ultrapure water for 30 minutes. In two separate reactions, 4 μl of the ligation mix were electroporated in 50 μl of super competent *E. coli* XL-1 blue cells (Agilent) at 1700 V, using the Electroporator 2510 (Eppendorf) and recovered in 1 mL pre-warmed SOC medium for 1 hour. Afterwards, 1 μl of from the recovery of each reaction was plated on a LB-Amp100 plate to determine the efficiency and coverage of the electroporation and the remaining two times 999 μl were used to inoculate a single flask with 150 mL of LB-Amp100 medium and grown overnight for maxiprep with the Nucleobond Xtra Maxi Kit (Macherey-Nagel).

## Nested sgRNA amplification protocol

To maintain a high library coverage of over 200-fold in the genomic PCR template, around 5 μg of genomic DNA was determined to be required as template per reaction. For robust amplification from such a high template amount, the Titanium Taq DNA Polymerase (Takara Bio) was used, following the pipetting scheme and thermos cycler program below:

Supplementary Table 3: Titanium Taq genomic PCR protocol

| **x1 (50 μl)** |  | **Program:** |  |  |
| --- | --- | --- | --- | --- |
| 5 μl | 10X Titanium Buffer | 95°C | 5 min |  |
| 0.5 μl | dNTPs (100 μM) | 95°C | 15 sec | 15 Cycles |
| 2.5 μl | oPS_0357 (10 μM) | 68°C | 30 sec |  |
| 2.5 μl | oPS_0358 (10 μM) | 72°C | 1 min |  |
| 1 μl | Titanium Taq Pol. | 72°C | 5 min |  |
| x μl | genomic DNA (5 μg) | 8°C | Hold |  |
| 38.5 μl - x | | nuclease free water |  |  |

To further increase the amplicon concentration with high specificity and proof-reading, the Herculase II Fusion Polymerase was used on ultrapure agarose gel-extracted fragments. Internally binding primers were used to specifically amplify the template for deep sequencing, as to see below:

Supplementary Table 4: Herculase PCR protocol

| **x1 (100 μl)** |  | **Program:** |  |  |
| --- | --- | --- | --- | --- |
| 10 μl | 10X Herculase Buffer | 95°C | 2 min |  |
| 1 μl | dNTPs (100 μM) | 95°C | 20 sec | 15 Cycles |
| 2.5 μl | oPS_0461 (10 μM) | 61°C | 20 sec |  |
| 2.5 μl | oPS_0462 (10 μM) | 72°C | 6 sec |  |
| 2 μl | Titanium Taq Pol. | 72°C | 3 min |  |
| 2 μl | Nested template (100 ng) | 8°C | Hold |  |
| 80 μl | | nuclease free water |  |  |

The PCR fragment was again cleaned up from an ultrapure 1% agarose gel, the DNA concentration determined with a NanoPhotometer (Implen) and the samples submitted for 250-bp paired end Illumina Sequencing (Novogene).

## RT-qPCR protocol

Briefly, 100 ng of RNA were converted to cDNA using the PrimeScript RT Reagent Kit (Takara Bio) and 5ng cDNA used as qPCR template with the Luna® Universal qPCR Master Mix (NEB) in triplicates and “-RT” control with the protocol depicted in Supplementary Table 5. The primers “CregA-9up” and “CregA-2down”, a kind gift from Dr. Anne Eugster (CRTD, Dresden) were used to amplify TRAC with the CFX96™ Real-Time PCR Detection System (Bio Rad) and primers “GAPDH_fwd” as well as “GAPDH_rev” were used to amplify GAPDH as a reference (Supplementary Table 7).

Supplementary Table 5: Luna qPCR protocol

| **x1 (10 μl)** |  | **Program:** |  |  | |
| --- | --- | --- | --- | --- | --- |
| 5 μl | Luna qPCR Master Mix | 95°C | 15 min |  | |
| 0.1 μl | fwd primer (10 μM) | 95°C | 20 sec | 40 Cycles |  |
| 0.1 μl | rev primer (10 μM) | 60°C | 20 sec |  |  |
| 2.8 μl | nuclease free water | 95°C | 1 min |  |  |
| 2 μl | cDNA or -RT control | 55°C | 15 sec | Melt Curve,  increment by 0.5 °C | |
|  |  | to 95°C | 15 sec |  |  |
|  | |  |  |  |  |

Analysis was performed by averaging the c(t) values of the technical triplicates for each sample. Thereafter, the ∆C(t) was calculated as the difference of the mean GAPDH c(t) and the mean TRAC c(t). From the biological duplicates of the untreated cells, the reference ∆C(t) was determined as average of both replicate’s ∆C(t). ∆∆C(t) was calculated as the difference of each sample’s ∆C(t) to the reference ∆C(t). The fold change was calculated as 2^-∆∆C(t) for each sample, its average and standard deviation were calculated between the biological duplicates and visualized.

# Supplementary Material

## sgRNA List

The complete list of all gRNA oligos in the pooled TRA library can be found in the separate supplementary file “sgTRA_Lib.xlsx”. Supplementary Table 6 lists only the sgRNAs, used in independent experiments.

Supplementary Table 6: sgRNA list

| **gRNA name** | **Spacer Sequence** | **Applications** | **Reference** |
| --- | --- | --- | --- |
| sgTRAC-1 | AGAGTCTCTCAGCTGGTACA | CRISPR TRAC Knockout | Present study |
| sgTRAC-4 | GGCTGTGGTCCAGCTGAGGTG | CRISPR TRAC-GFP Knockin | Present study |
| sgTRA-1 | AGCTGGCTGATGTAGCTCAC | TCRA promoter silencing | Present study |
| sgTRA-357 | CATAGTTCCTTTGCTGGGAA | Hit validation (neg. c.) | Present study |
| sgTRA-505 | ACAAAACTGTGCTAGACATG | Hit validation (pos. c.) | Present study |
| sgTRA-743 | GATTAAACCCGGCCACTTTC | Hit validation (pos. c.) | Present study |
| sgTRA-100 | GGGCAGCTTAGAAAGAACTG | Hit validation | Present study |
| sgTRA-826 | GGCAGTGACAAGCAGCAATA | Hit validation | Present study |
| sgTRA-1040 | GAGCCTGGACAGCAGTGAAG | Hit validation | Present study |
| sgTRA-1289 | TTCTGCGGGAGAGCTTCAAA | Hit validation | Present study |
| sgTRA-1291 | CCCGCAGAAGCCACATCCTC | Hit validation | Present study |
| sgTRA-1813 | CAGAAACGCAATGGGGCTTG | Hit validation | Present study |

## Oligo List

Supplementary Table 7: Oligo List

| **gRNA name** | **Sequence** | **Application** | **Reference** |
| --- | --- | --- | --- |
| oLi_596 | TTTTTTTTTTTTTTTTGGTTTATTCCCCCGTTTAAACTCATTACTAACCGGT | mRNA template PCR | Present study |
| oLi_921 | GCTAATACGACTCACTATAGGGAACATTTGCTTCTGACACAACTGTGTTC | mRNA template PCR | Present study |
| oPS_0159 | CTGCTCTGCCTTGGGGAAAA | Genotyping PCR “full” | Present study |
| oPS_0225 | CAACTGAGGCGGCTGAAATG | Genotyping PCR “full” | Present study |
| oPS_0226 | GCTCCACTGTCAAGAGAGCC | Genotyping PCR “left flank” | Present study |
| oPS_0228 | TGTGGTAGGTGCCGTTCTTC | Genotyping PCR “left flank” | Present study |
| oPS_0229 | GTCTGTGCTGAAGCCATTGC | Genotyping PCR “right flank” | Present study |
| oPS_0230 | GGCTCTGTTTGGGGCTATGT | Genotyping PCR “right flank” | Present study |
| oPS_0273 | GATATTGCAACGTCTCACACC | Library amplification | Present study |
| oPS_0274 | GTCGCGTACGTCTCGAAAC | Library amplification | Present study |
| oPS_0357 | GCGCCAAAGTGGATCTCTGCTGTCCCT | gRNA genomic PCR | Present study |
| oPS_0358 | ACCATTATCGTTTCAGACCCACCTCCCA | gRNA genomic PCR | Present study |
| oPS_0522 | aaCGTCTCAcaccACCCAGTGCTGTCATTCTGG | Subcloning of Ea Alleles | Present study |
| oPS_0523 | ttCGTCTCAaaacGTTTGGAGGGGCAGCAGAG | Subcloning of Ea Alleles | Present study |
| oPS_0520 | GTCTGTGTCAAGGCCACCTG | Ea Sanger Seq. | Present study |
| CregA-9up | CGCCTTCAACAACAGCATT | RT-qPCR of TRAC | Present study |
| CregA-2down | AGATTAAACCCGGCCACTTT | RT-qPCR of TRAC | Present study |
| GAPDH_fwd | ACCCAGAAGACTGTGGATGG | RT-qPCR of GADPH | Present study |
| GAPDH_rev | TTCAGCTCAGGGATGACCTT | RT-qPCR of GADPH | Present study |
